# A Triad of Ketoacidosis, Hypertriglyceridemia, and Acute Pancreatitis Associated With Sugar-Sweetened Soft Drinks Abuse in a Caucasian Patient With Undiagnosed Type 2 Diabetes Mellitus

**DOI:** 10.7759/cureus.8299

**Published:** 2020-05-26

**Authors:** Alessandra Vitelli, Valentina Apuzzi, Francesco Calderaro, Olimpia Fattoruso, Vincenzo Bassi

**Affiliations:** 1 U.O.C. Medicina Generale E Lungodegenza, San Giovanni Bosco, Asl Napoli 1 Centro, Naples, ITA; 2 U.O.C. Medicina Genereale E Lungodegenza, San Giovanni Bosco, Asl Napoli 1 Centro, Naples, ITA; 3 Pathology, San Giovanni Bosco Hospital, ASL Napoli 1 Centro, Naples, ITA; 4 U. O. C. Di Medicina Generale E Lungodegenza, San Giovanni Bosco Hospital, Asl Napoli 1 Centro, Naples, ITA

**Keywords:** diabetes mellitus, ketoacidosis, hypertriglyceridemia, acute pancreatitis, soft drinks, western patient

## Abstract

A 24-year-old obese Caucasian male, without relevant anamnesis, who was admitted to the ER presented with abdominal pain, nausea and vomiting, hyperglycemia, and diabetic ketoacidosis (DKA). The diagnosis of acute pancreatitis (AP) was supported by increased serum levels of triglycerides and lipase associated with abdominal CT scans. The patient was treated for five days with IV regular insulin, hydration, electrolytes replacement, and statin/fibrate therapy with clinical improvement. Some 10% hemoglobin A1c value, normal C-peptide level and negative glutamic acid decarboxylase (GAD-65), and islet cell autoantibodies suggested the diagnosis of a new-onset type 2 diabetes mellitus (DM) presenting with an uncommon triad of DKA and hypertriglyceridemia (HTG)-induced AP. Anamnestic history suggested that DKA was dependent on sugar-sweetened soft drinks abuse (soft drink ketosis), a clinical association more frequent in Asian than in Western patients.

## Introduction

Diabetic ketoacidosis (DKA) is an emergency condition characterized by hyperglycemia, metabolic acidosis, and hyperketonemia. Usually, DKA is secondary to an absolute insulin deficiency typical of type 1 diabetes mellitus (DM); however, DKA could be rarely seen in patients with type 2 DM when a relative pancreatic β cell dysfunction occurs [1-4]. Insulin deficiency induces both glucose and lipid metabolism dysregulation, such as hyperglycemia and hypertriglyceridemia (HTG) [5-7]. Usually, serum triglycerides (TG) value > 1000 mg/dL is responsible for four percent of overall acute pancreatitis (AP) secondary to toxic free fatty acids (FFA) from TG breakdown [8-9]. Moreover, in Eastern countries, an increasing number of case reports of DKA from both type 1 and type 2 DM are described as secondary to sugar-sweetened soft drinks overconsumption (soft drink ketosis) [6, 10-13].

## Case presentation

A 24-year-old obese Italian male (body mass index = 48.9 kg/m2) was admitted to our ER Unit presenting with abdominal epigastric pain and persistent nausea with vomiting in the last four days. Medical history was negative to alcohol abuse, cholelithiasis, dyslipidemia, or drug therapy; however, familiar history of hypertriglyceridemia (mother and maternal grandfather) was reported. Four liters daily intake of sugar-sweetened soft drinks was referred. On clinical examination, the patient presented with severe abdominal pain without epigastric resistance and negative Murphy’s sign, tachyarrhythmia (heart rate=120 beats/min), hyperthermia (38°C), severe dehydration, and Kussmaul breathing. Laboratory parameters clearly showed metabolic acidosis, hyperglycemia, increased Hb A1c value, and severe HTG resulting in lactescent serum. Normal serum values of C-peptide, glutamic acid decarboxylase (GAD-65), and islet cell autoantibodies suggested the diagnosis of new-onset type 2 DM (Table 1). Abdominal CT scans showed an edematous AP. The patient’s DKA was treated for five days with regular insulin IV infusion associated with aggressive fluid replacement starting with normal saline solution, then a 10% glucose solution after a gradual decrease of glycemia and potassium supplementation [14]. Fenofibrate (145 mg orally, twice a day) and atorvastatin (20 mg once a day) were started obtaining a fast HTG decrease (<500 mg/dL in about 72 hours) excluding the need of plasmapheresis. A gradual decrease of serum glucose levels, ketones, and TG were observed (Table 2). A low-calorie diet was introduced and insulin IV therapy was interrupted, starting a basal bolus insulin regimen when hyperglycemia, metabolic acidosis, and hyperketonemia were corrected and epigastric pain disappeared. CT scan and serum lipase values normalized in about 45 days. The patient was discharged on the 23rd day with basal-bolus insulin regimen, metformin, atorvastatin, fibrates, omega-3 therapy suggesting a fat-restricted diet.

**Table 1:** Initial laboratory data.

Laboratory parameters	Values
HbA1c (<6.5 %)	10.9
Serum glucose (60-110 mg/dL)	324
C-peptide (0.9-7.1 ng/mL)	4.7
Glutamic acid decarboxylase (GAD-65) autoantibodies (<5 U/mL)	<5
Islet cell autoantibodies (<1:4)	<1:4
Amylase (28-100 UI/L)	80
Lipase (13-60 UI/L)	554
Total cholesterol (<200 mg/dL)	596
Triglycerides (<200 mg/dL)	4797
Ketonemia (<0.6 µmol/L)	4.5
Uric acid (3.4-7.0 mg/dL)	10.9
Na (135-148 mEq/L)	131
K (3.6-5.3 mEq/L)	4.7
WBC (4.000-11.000/mm)	18500
Micro albuminuria (<1.9 mg/dL)	18.39
PCR (<0.5 mg/dL)	48,7
Ph (7.35-7.45)	7.27
SPO2 (>90 %)	99
PaCO2 (35-45 mmHg)	14
PaO2 (80-100 mmHg)	88
HCO3- (22-26 mmol/L)	6
Base excess (>−2 < +2 mmol/L)	−20.9
Lactate (0.3-1.2 mmol/L)	0.8
Anion gap	22

 

**Table 2:** Time course of biochemical data.

Lab test	At admission	24 h	48 h	72 h	120 h	At discharge (23rd day)
Serum glucose (60-110 mg/dL)	324	272	238	227	170	108
Ketonemia (<0.6 µmol/L)	4.5	3.4	4.2	3.6	0.5	0.2
Triglycerides (<200 mg/dL)	4797	746	628	431	314	272

## Discussion

Diabetic ketoacidosis results from insulin deficiency, increased lipolysis, and decreased activity of the lipoprotein lipase enzyme in the capillary endothelial cells of the adipose tissue. As a consequence, elevate circulating triglycerides can generate a higher concentration of FFA which contributes to pancreatic cell injury triggering AP [8]. It is important to consider that abdominal pain is common in severe DKA and serum amylase and lipase may show false-normal values in hyperlipemic serum or increased levels in 16%-25% of DKA patients without AP involvement [5,15]. Thus, physicians have to suspect AP in the presence of lactescent serum and persisting abdominal pain confirming the pancreatic damage with radiological images [16]. On admission, our patient presented many risk factors such as obesity, familiar hypertriglyceridemia, sedentary lifestyle, and excessive sugar-sweetened soft drinks daily intake. Classical triad of DKA-HTG-AP was confirmed, a rare evidence described with 11% prevalence in overall AP patients, unfortunately associated with higher rate of inpatient mortality, multiorgan failure, parental nutrition requirement, hospital charges, and longer hospital length of stay [17]. Moreover, the association of negative autoimmune markers and good β-cell function assigned a 54% probability of developing ketones in our patient [3]. Thus, prevention of the next episode, once HTG-induced AP episode has been resolved, is mandatory [18]. In recent years, the role of soft drinks consumption in glucose dysregulation has emerged in the Asiatic population with evidence of pancreatic β cells dysfunction correlated with prediabetes, DM, and DKA (soft drink ketosis) [6,10-13,19]. Tanaka et al. have suggested that a polymorphism in the β-3-adrenergic receptor gene (Ttrp64 homozygosis), involved in lipolysis, would be strictly associated with soft drink ketosis [11]. Currently, no evidence between soft drink ketosis and Caucasian patients has been found.

## Conclusions

We reported the case of a Caucasian obese patient with two uncommon characteristics such as a rare triad of DKA-HTG-AP and soft drinks abuse, a metabolic condition more typical in patients from Asia compared to Western countries origin. Thus, physicians have to discourage soft drinks abuse in patients presenting with DM risk factors or diagnosis. Lifestyle changes consisting of dietary modifications and physical activity, beyond drug therapy, are the key behind successful management of DM and hyperlipidemia. 
